# Implicit Theory of Mind under realistic social circumstances measured with mobile eye-tracking

**DOI:** 10.1038/s41598-020-80614-5

**Published:** 2021-01-13

**Authors:** Louisa Kulke, Max Andreas Bosse Hinrichs

**Affiliations:** 1 Department of Affective Neuroscience and Psychophysiology, Georg-August University Göttingen, Goßlerstr. 14, 37073 Göttingen, Germany; 2 Friedrich-Alexander-Universität Erlangen-Nürnberg, Erlangen, Germany

**Keywords:** Psychology, Human behaviour

## Abstract

Recently, there has been a debate whether implicit Theory of Mind can be reliably measured using anticipatory looking tasks. Previous anticipatory looking paradigms used video stimuli to measure implicit Theory of Mind; however, numerous replications of these paradigms were unsuccessful. This lack of replications may be due to video stimuli not being sufficiently engaging. As Theory of Mind is an inherently social phenomenon, robust evidence might only be observed in a real social situation. Therefore, the current preregistered study aimed to test anticipatory looking with real-life social stimuli. A mobile eye-tracker was used to measure gaze patterns indicative of Theory of Mind while participants observed a real-life interaction of an experimenter and a confederate. The realistic scenario did not provide clear evidence for implicit Theory of Mind. Furthermore, anticipatory looking behavior did not reliably occur during familiarization trials, in line with previous research. However, looking patterns were slightly more in line with belief tracking than in some more controlled studies using video stimuli. In general, implicit Theory of Mind was not reliably reflected in anticipatory looking patterns even if they were measured in realistic social situations. This questions the suitability of anticipatory looking measures for implicit Theory of Mind.

## Introduction

Theory of Mind (ToM) has recently become a hot topic of discussion^1–3^. This phenomenon is defined as the ability to impute mental states to oneself and others^4^, such as beliefs, desires and intentions. Some studies found evidence that people attribute mental states to others implicitly. These studies suggest that this skill develops early during development, and that implicit ToM can be measured with non-verbal measures, including *violation of expectation* looking time paradigms^5,6^, *interactive behavioral tasks*^7–9^, *priming*^10–12^ and *anticipatory looking* measures [e.g.^13–18^]. This implicit, automatic form of ToM has been found to remain stable across the lifespan^13,19,20^ and in particular anticipatory looking paradigms can be and have been used in exactly the same way across the lifespan [see e.g.,^14,15^].

However, a growing number of non-replications questions the reliability of these implicit ToM findings [for an overview, see^21^]. Numerous studies failed to replicate violation of expectation paradigms^22–24^, interaction tasks^25,26^ and anticipatory looking tasks [e.g.^27–35^]. In particular, non-replications of anticipatory looking tasks are surprising. In studies that use these tasks, participants are typically presented with recorded videos, in which a first agent (agent 1) hides an object in one of two boxes while a second agent (agent 2) observes the hiding process. Without agent 2 watching, agent 1 subsequently either transfers the object to the second box or removes the object from the scene, leading to agent 2 having a false-belief about the object’s location. The tasks are also known as “change-of-location false-belief-task”^8,14,15,36^. Participants’ looking in anticipation of where agent 2 will search for the object is measured. It is reasoned that if participants have an implicit ToM, they should anticipatorily look to the location where agent 2 will incorrectly search for the object, reflecting her false belief. Replication studies often use identical stimulus videos as the original tasks and desk-mounted eye-tracking as an objective measure. In contrast to non-replications of other paradigms this leaves little room for subjective or researcher-related factors to influence the findings^1,2,29,32^. In addition, non-replications of anticipatory looking paradigms are troubling because these paradigms have been used across the human lifespan and resulted in far reaching conclusions about a lifelong capacity of implicit ToM that was suggested to exist next to an explicit, verbal reasoning system [see e.g.,^8,15^]. With non-replications affecting virtually all anticipatory looking paradigms and all age groups there is currently a debate on how to interpret non-replications^1,2^. Replications may either fail because implicit ToM does not exist or because it is difficult to measure and previous tasks have not been sufficiently sensitive to find reliable evidence^34^.

To clarify the confusing pattern of original findings, partial and non-replications, systematic studies have been conducted to investigate under which conditions implicit ToM tasks can be replicated. Confounds in original studies using video stimuli, like cueing effects due to turning of the agent or due to the object always ending up at the same location have been identified. If these confounds are removed, replications fail consistently^32,33^. Another factor which may influence the replicability of implicit ToM measures is that the video stimuli are not very engaging, but often present simple environments in which a hand puppet and an actress interact by moving a ball between two boxes. When more realistic stimulus videos with engaging chase scenarios are used, anticipatory looking in line with ToM could be demonstrated in apes, for whom previously no ToM could be observed^37^. However, a recent attempt to use comparably engaging chase scenarios in humans did not provide evidence for implicit ToM^35^. This suggests that the stimulus materials might need to be designed in an even more realistic fashion for humans. When comparing the replicability of different paradigms, the paradigm by Clements and Perner^18^ seems to replicate most reliably^21^. This paradigm differs from other, less replicable paradigms in two ways. Firstly, it uses a verbal narration of the events instead of only presenting a non-verbal video. Recent attempts to combine other video-based paradigms^14,15^ with verbal narration did however not provide evidence for implicit ToM^34^. Secondly, the paradigm by Clements and Perner^18^ uses a more realistic scenario in which participants are not presented with video stimuli but the experimenter interacts with participants and live-enacts the false belief task using a cardboard scenario. A higher engagement of this live procedure may facilitate belief tracking, leading to more reliable anticipatory looking in line with ToM. In fact, recent research suggests that live interactions fundamentally differ from videos^38–40^. The paradigm of Clements and Perner^18^ is only suitable for testing children, as a game-like pretend play scenario is enacted. However, to our knowledge, anticipatory looking has not been measured in response to real-life interactions between two people, which would be suitable for testing adults as well as children. In the debate of whether implicit ToM exists, testing adults is of increased theoretical value. In contrast to (pre-verbal) infants it can be assumed that adults have a fully developed ToM as shown in studies using explicit tasks^35^ and as indicated by our everyday life experience. Adults continuously engage in mind reading during everyday life. In contrast, non-replications with (pre-verbal) infants, may be explained by the fact, that ToM has not yet fully developed, rather than indicating that implicit ToM does not exist at all.

Corresponding to the findings of Clements and Perner, recent evidence from real-world eye-tracking studies suggests that gaze behavior differs between participants viewing video stimuli compared to gaze behavior during real social situations^41–43^. Automatic belief-tracking might be more robustly elicited in life situations compared to video watching since there is a potential for interaction, and uncertainty whether knowing what another protagonist thinks, knows or beliefs, might become important in the future. In fact, recent research suggests that brain areas involved in the mentalising network are activated when participants believe to be in a life situation, even if no interaction occurs^44^. In contrast, when watching video stimuli, constant, automatic belief tracking is not ultimately necessary, since participants know beforehand, that there will not be a real interaction and that the situation is comparably predictable. Therefore, knowing the other’s mental states does not have interactional relevance in the context of video stimuli but it does in the context of a real social interactions. As ToM is an inherently social phenomenon^45^, robust evidence for anticipatory looking implicit ToM might only be obtained in a real social situation. Therefore, the aim of the current study was to develop a more realistic interactive paradigm in which anticipatory looking can be measured, that is suitable for testing adult participants. For this purpose, adults’ gaze behavior was measured while participants watched an interaction of an experimenter with a confederate in a live social situation, instead of watching a video as in previous studies. If implicit ToM is difficult to measure but exists, it may reveal itself more clearly in this realistic situation. Therefore, we predicted anticipatory looking behavior in line with ToM. A further problem with previous video-based anticipatory looking measures, is the high dropout rate in original^15^ and replication studies^26,29,30,32,33,35^, in some of which more than 50% of the sample are excluded, due to participants not even showing anticipatory looking during familiarization trials. The lack of any anticipatory looking (the main measure of these tasks) questions the validity of the tasks. Participants may not be sufficiently engaged by video-based tasks to show reliable anticipatory looking at all. Presuming that the lack of overall anticipatory looking reflected in high dropout rates in video-based tasks was due to low engagement, we predicted lower dropout rates in the current compared to previous replication studies, as real-life interactions should engage attention more clearly. The paradigm of Southgate, Senju^15^ and Senju, Southgate^36^ was used as a basis for conceptual replication. However, the current study removed confounds identified by previous studies (i.e., the turning direction of the actress and cueing due to the object being always hidden in the same final location) to avoid visual effects rather than belief tracking affecting the observed gaze pattern.

In summary, the current study aimed to investigate, firstly, whether robust evidence for false belief understanding measured through anticipatory looking can be found in a real-life situation, and, secondly, whether drop-out rates are decreased in realistic situations compared to video-based paradigms.

## Method

### Participants

The current study was preregistered with the Open Science Framework (https://osf.io/ntj2y). To reach the predetermined sample size of 52 included participants, *N* = 100 participants (age range: 18–35 years, *M*_age_ = 23.97 years, *SD*_age_ = 3.24 years, 55 females) were tested. Of these participants, two were first-year psychology students who had not yet been introduced to “Theory of Mind” during their lectures. Neither of the psychology students guessed the aim of the study during debriefing. Participants were excluded from data analysis if they did not meet the familiarization criterion defined by Senju et al. (2010, i.e., longer looking to the correct area of interest (AOI) in the last familiarization trial, *n* = 40, these participants were only included in the dropout analysis), if they did not show a first look to one of the two AOIs in the first false-belief test trial (*n* = 6), due to technical (*n* = 1) or experimenter error (*n* = 1). Additional *n* = 7 participants were invited to the test session but did not begin the test session, as the mobile eye-tracker could not record their gaze reliably. Due to the use of a mobile eye-tracking headset, people with glasses could not participate. People with visual impairments were tested if they corrected their vision using contact lenses. All participants were native German speakers and recruited via posters, leaflets, email, or verbal recruitment on Göttingen campus and reported to have no psychological or neurological disorder. Testing took approximately half an hour and participants received 4 € or 0.5 course credit in return for participation. The study was conducted in line with the Declaration of Helsinki and reviewed by the local ethics committee of the Göttingen University Psychology Department. Participants’ written consent was collected before testing and after the full debriefing about all deceptions used which was conducted at the end of the study. Written consent for publication of identifying images was obtained from the confederate and the experimenter.

### Task

The current task was closely modeled on the original anticipatory looking false belief task by Southgate, Senju^15^ and Senju, Southgate^36^, with the major difference being that instead of watching a video, participants observed a live interaction. In the original video stimuli, two agents interact. Agent 1 puts an object into a box and agent 2 observes the hiding, standing behind a panel with two windows to reach through. Agent 2 retrieves the object from the box by reaching through one of the windows upon a sound cue and the simultaneous illumination of the two windows during familiarization trials. The familiarization trials familiarize participants that agent 2 is attentive and motivated to retrieve the object after the sound cue. Subsequently, during two test trials, agent 2 watches agent 1 put an object into a box. In the presence (false-belief condition 1, FB1) or absence (false-belief condition 2, FB2) of agent 2′s attention, agent 1 then moves the object to a second box. Agent 1 subsequently removes the object from the scene while agent 2 is not looking. Both conditions result in agent 2 having a false belief about the location of the object, since agent 2 has not seen the object being removed. However, the belief congruent location either is identical to the last object location (in the FB1 condition, i.e., the first box) or differs from it (in the FB2 condition, i.e., the second box). Participants’ anticipatory gaze in expectation of where agent 2 will look for the object is measured in the time window following the sound cue onset. The combination of the FB1 and the FB2 condition controls for the possibility of mere object tracking. Only if participants show correct action anticipation in both conditions (FB1 and FB2), this would construe clear evidence for belief-tracking, since for “correct” anticipation in the FB1 condition, participants could merely look to the last object location without engaging in belief-tracking. Due to the realistic nature, in the current study, no windows were illuminated but only the original sound cue was played. While in the original study a ringing telephone distracted agent 2′s attention, in this study a blind was introduced. In the current study, the change-of-location task from the original videos was live enacted.

### Procedure

**Figure 1:**
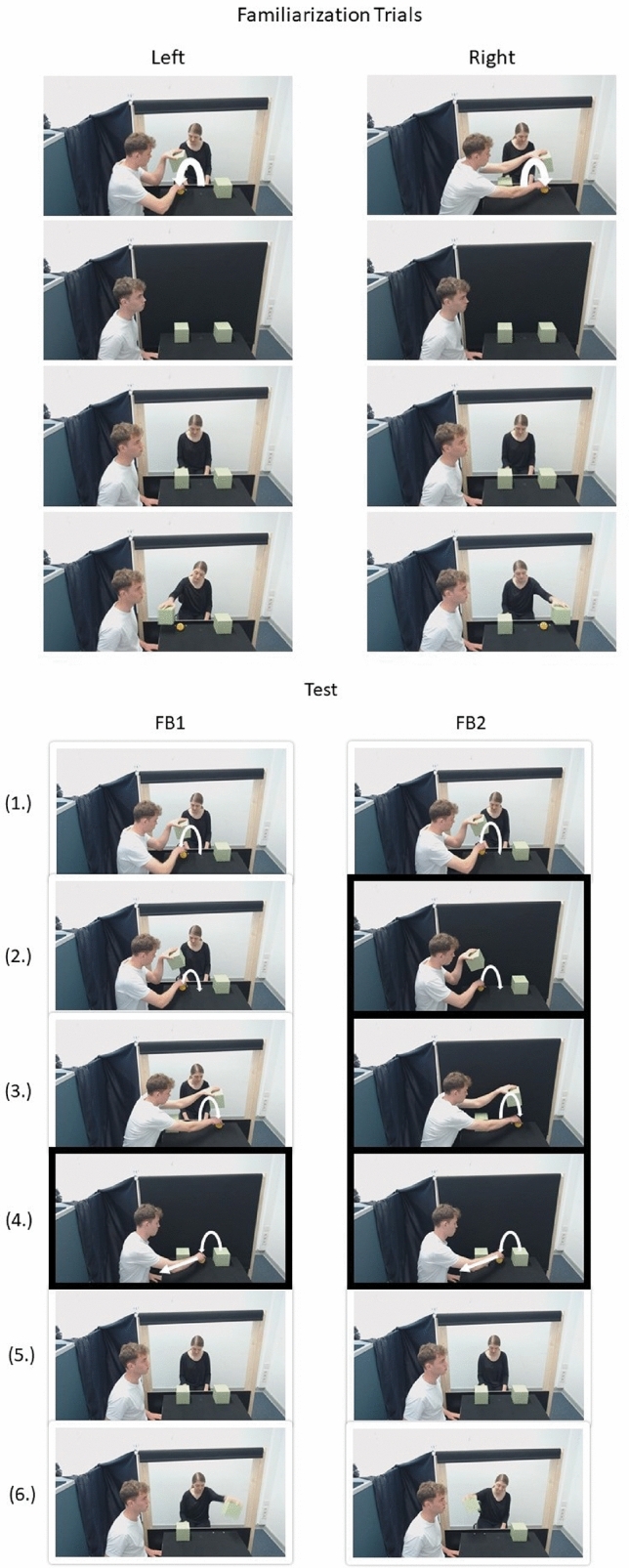
Order of behavior of the experimenter (left person) and the confederate (central person) during the familiarization and test trials. The FB1 and FB2 conditions differ regarding when the blind is closed (indicated by a black box around the images) or open.

Upon arrival, participants were told the cover story that the study was about play behavior in social contexts and that the confederate was another participant. Both the participant and the confederate were instructed by the experimenter that they would be involved in a game during which one person’s task was to watch him hide a ball under one of two boxes, remember the location of the ball and retrieve it after a cue sound. The instructions stated that the other person’s task was to observe the game while wearing a mobile eye-tracker. The experimenter stated that they would randomly be assigned to the two tasks, but always assigned the participant to the observer’s task and the confederate to the active task. The experimenter then fitted the mobile eye-tracker to the participant, calibrated it (see below) and started the experiment. Screenshots of the conditions are displayed in Fig. 1. Details regarding the set up are depicted in Supplementary Information A. Firstly, during four familiarization trials, the experimenter placed a ball under one of two boxes (starting with the left or right side in a counterbalanced fashion and alternating between both sides, i.e., in the order left–right–left–right or right–left–right–left). Note that in the original studies, all participants received familiarization trials in the order left–right–left–right. In this study the order of familiarization trials was counterbalanced across participants. The experimenter closed a blind so that the confederate’s view was completely obstructed from the scene. He reopened the blind after 10 s (silently counted by the experimenter) and activated a cue sound. The confederate waited for 3 s (silently counted) until reaching for the ball with the hand corresponding to the correct box. Subsequently, participants completed two test trials. In the original video stimuli, the object was initially placed under the left box in both false belief conditions. In this study, the initial hiding location was counterbalanced between participants. Depending on the order of familiarization trials both test trials either started with the experimenter placing the ball under the left or under the right box, so that the two possible orders counterbalanced between participants where right–left–right–left–right–right or left–right–left–right–left–left. The sequence acted out in the current study is displayed in Fig. 1. In the FB1 condition, the experimenter (1.) placed the ball under one box, (2.) removed it from the box and (3.) moved it to the other box. He then closed the blind, (4.) removed the ball from the scene and (5.) re-opened the blind. Subsequently, a cue sounded and (6.) the confederate reached for the belief-congruent box 6 s (silently counted) after the cue. In the FB2 condition, the experimenter (1.) placed the ball under one box. However, he now already closed the blind, (2) removed the ball from the box and (3.) moved it to the other box, (4.) removed it from the scene, and (5.) re-opened the blind. As in the FB1 condition, subsequently, a cue sounded, and (6.) the confederate reached for the belief-congruent box 6 s (silently counted) after the cue. Participants’ anticipatory looking behavior to the left and right side was measured within the interval of 6 s between cue sound and reaching of the confederate. To control for gaze cueing effects, during and upon the reopening of the blind, the confederate kept central fixation, sat still and did not move her head until she had lifted the respective box in all trials. The order of the FB1 and FB2 condition was counterbalanced between participants. The confederate was blind to the order each participant completed the task in. Having lifted the belief-congruent box and not finding the ball in the first false-belief trial, the confederate looked at the experimenter smiling at which point the experimenter looked at the confederate and said “alright I’ll prepare it again”. The experimenter subsequently closed the blind and placed the object centrally between the two boxes. The experimenter then reopened the blind and started the second false-belief trial. To ensure comparability of conditions, the confederate and experimenter abided by a strict behavioral protocol and their behavior was filmed with two webcams to ensure that the protocol was followed. Timing was trained in advance.

Upon completion of the change-of-location task, both the participant and the confederate filled in a debriefing questionnaire [based on^13^, assessing whether participants were aware of the aim of the study and/or the presence of the confederate and whether observing the change-of-location task they used any strategy.

### Technical equipment and eye-tracking

A mobile binocular Pupil Labs eye-tracking headset^46^ was used to record gaze data, consisting of two 200 Hz eye cameras and a 120 Hz world camera that recorded the visual scene. Eye-tracking data was recorded at an average sampling rate of 26.3 frames per second (*SD* = 2.57). The eye-tracking headset was connected to a Dell Latitude E5530 computer (operating system: Windows 8.1 Enterprise, processor type: Intel Core i5-3210 M 2.50 GHz, RAM: 8 GB, system type: 64-bit-operating system) via USB. Pupil Capture software (release 1.10, January 2019) was used to control calibration and recording. Audio was recorded via a computer inbuilt microphone using the audio capture plugin of the Pupil Capture software. Following the Pupil Labs recommendations for mid-range viewing distances, a 9-point manual marker calibration was used to calibrate the eye-tracking device before testing. For this purpose, participants were asked to fixate a printed calibration marker. The experimenter held the marker in a grid-like manner, at approximately nine locations in the participant's relevant field of view^47,48^. Calibration quality was evaluated through a validation procedure, in which the experimenter asked the participant to fixate the marker again at nine points and then used the live video feed of the Pupil Capture software to check for coherence between the depicted gaze position and the depicted position of the marker^47,48^. Calibration was accepted, once clear coherence between the gaze position and the position of the marker was observed at the four relevant locations for this study (i.e., the location of the two boxes, the ball location central between the two boxes and the location of the confederate’s head).

### Design

Anticipatory looking was measured as, firstly, the direction of the first saccade towards the belief-congruent or belief-incongruent side and, secondly, as the looking time to the belief-congruent minus the looking time to the incongruent side divided by the overall looking time to both sides (differential looking score, DLS)^14^. Because many participants did not show a first saccade, we also analyzed first looks as a more lenient measure to increase statistical power (see “Eye-tracking data reduction” section). Note that the analysis of first looks was exploratory and not preregistered. In the original studies, FB1 and FB2 conditions were compared between participants. In the current study each participant completed both conditions in counterbalanced order in a within-participant design to increase statistical power. A separate between-participant analysis of first trials was conducted to model the between-participant design of the original studies^26,30^. Dropout rates due to participants not showing correct anticipatory looking in the familiarization trials were compared between studies.

### Eye-tracking data reduction

In the original study of Senju, Southgate^14^, two windows were used as AOIs for the coding of first saccades and the computation of the DLS. As the current study used a more realistic set up without a panel and windows, the two boxes and the area surrounding the boxes, including the arms of the confederate, were used as AOIs (see Supplementary Information B for a detailed description and graphical illustration of the AOIs). Note that a similar approach with boxes as AOIs has been taken by Schneider, Bayliss^13^, Kulke, Johannsen^33^ and Kulke, Wübker^35^.

After data collection, Pupil Player software (release 1.10, January 2019, default settings) was used to playback eye-tracking recordings for data reduction. The sound cue onset for a respective trial was identified manually by two independent coders. Gaze behavior was examined for 3 s, starting at sound cue onset in the fourth familiarization trial and for 6 s starting at sound cue onset in false-belief test trials (FB1, FB2). The analysis interval of 6 s was based on the analysis interval of the original study^14^. The analysis interval of 3 s was also based on this study. In the two original video stimuli the actress waited for approximately 3 s after sound cue onset in the last familiarization trial and anticipatory looking was measured during this interval. We chose a time window of 3 s in order to allow the confederate to internally count the appropriate interval. The direction of the first saccade was coded as the first eye-movement in the direction of one of the two AOIs after sound cue onset. If a participant already looked at one of the two AOIs at sound cue onset, this was classified as a first look instead. Note that the first look measure is partially redundant with the first saccade measure. The first saccade measure is more conservative, as it excludes the possibility that participants’ gaze still lingers on an AOI due to the previously observed action rather than due to anticipation. However, the first look measure allows inclusion of more participants, potentially providing more power. It was classified whether the respective saccade or look was correct (i.e., directed towards the location congruent with the confederate’s belief) or incorrect (i.e., directed towards the belief-incongruent location). Interrater agreement for two independent coders (one of whom was author MH) computed across 50% of all trials was high (90.37% for whether a first saccade occurred, 88.89% for whether this first saccade was correct, 94.81% for whether a first look occurred and 94.81% for whether this first look was correct). Based on the formula provided by Senju, Southgate^14^, the DLS was calculated in MATLAB (R2017a, MathWorks) as the total looking time to the belief-congruent AOI minus the total looking time to the belief-incongruent AOI, divided by the sum of looking time towards both AOIs (congruent and incongruent). To obtain the looking times to each AOI, two independent coders coded frame-by-frame where a participant gazed during the respective analysis intervals. Interrater agreement for two independent coders (one of which was author MH) was 98.41% (calculated across 50% of all trials).

### Statistical analyses

#### Confirmatory analyses

Two-tailed tests and a significance level of 0.05 were used for all analyses. In accordance with the preregistration, null-effects were followed up with Bayesian statistics. To analyze the anticipatory looking measures (first saccades, first looks, DLSs), in a first step, a between-participant analysis was conducted considering only the first false-belief trial for each participant. Subsequently, in a second step, a full-set analysis was conducted, including the first and the second false-belief trial [comparable to^26,30^]. For both, the first trial and the full-set analyses, binomial tests were used to examine whether the number of correct first saccades significantly differed from chance (0.5) in each condition (FB1, FB2). For the first trial analysis, Fisher’s exact test was used to examine whether the number of correct first saccades differed between the two conditions (FB1, FB2). For the full-set analysis, McNemar’s test was used to investigate whether the number of correct first saccades differed between the two conditions (FB1, FB2) within participants. The same statistical tests that were conducted on first saccades were conducted on first looks. Note that the statistical analyses on first looks were not preregistered but provide more power. For both the first trial and the full-set analyses, one-sample t-tests examined whether the mean DLS significantly differed from zero in each condition (FB1, FB2). For the first trial analysis, an independent t-test examined whether the mean DLS significantly differed between the two conditions (FB1, FB2). For the full set analysis, a dependent t-test tested whether the DLS significantly differed between the two conditions (FB1, FB2) within participants.

The dropout rate due to participants not meeting the familiarization criterion defined by Senju, Southgate^36^, i.e., longer looking to the belief-congruent AOI in the last familiarization trial, was compared to previous studies using binomial tests. The dropout analysis was based on the approach of Kulke, Wübker^35^ who compared the number of participants that were overall included in the data analysis with the number of dropouts due to not passing the familiarization criterion. For comparability, the dropout rate was compared to the same studies as in the study of Kulke, Wübker^35^ [i.e.^15,36^]. Because Kulke, Wübker^35^ tried to enhance the ecological validity of video stimuli, the dropout rate was also compared to their studies (study 1 and study 2), to have a direct comparison between more realistic video stimuli and the real life situation used in this study.

#### Exploratory analyses

It was preregistered that the effect of whether participants guess the aim of the experiment during the debriefing procedure on the observed results would be explored. This effect was explored descriptively because of the small number of participants (*n* = 2) who guessed the aim.

The mean DLSs and the proportions of correct first saccades in this study were compared to previous replication studies of the Southgate/Senju paradigm that tested adult participants. Specifically, the results were compared to the studies of Kulke, Johannsen, et al. (2019, study 1 and study 3), the study 2b of Kulke, Reiß, et al. (2018), the study of Kulke, von Duhn, et al. (2018) and the studies of Kulke, Wübker, et al. (2019, study 1 and study 2). The DLSs were compared using one-sample t-tests. The proportions of correct first saccades were compared using binomial tests. Note that these analyses were exploratory and not preregistered.

#### Analysis packages

All statistical analyses were conducted using the programming platform R [RStudio version 1.1.463;^49^]. The analysis script used for this study can be found in Supplementary Information C, the full dataset is available in Supplementary Information D. Binomial tests (function: *binom.test*), Fisher’s exact tests (function: *fisher.test*), McNemar’s tests (function: *mcnemar.test*), and t-tests (function: *t.test*) were conducted using the *stats* package [version 3.5.2;^49^]. Note, that McNemar’s tests were computed with continuity correction (as part of the default settings of the mcnemar.test function). For the number of correct first saccades and first looks, the probability of success and its confidence interval are reported in the results section. These values were taken from the output of the binom.test function in R. The odds ratio effect size (OR) that is reported for fisher’s exact tests was extracted from the output of the fisher.test function in R. Cohen’s *d* effect size was computed using the function *cohensD* of the *lsr* package [version 0.5;^50^]. The function *glmer* of the *lme4* package [version 1.1–21;^51^] was used to compute generalized linear mixed-effects models. Follow up Bayesian analyses for all statistical tests, except for the McNemar’s test were computed using the *BayesFactor* package [version 0.9.12–4.2;^52^]. The function *proportionBF* was used to follow-up binomial tests, the function *contingencyTableBF* (sample type “poisson”) was used to follow up fisher’s exact tests, and the function *ttestBF* was used to follow-up t-tests. To date, no direct Bayes factor equivalent for the McNemar’s test is implemented in the BayesFactor package. Therefore, the respective Bayes factor for the within-participant comparisons of correct first saccades and correct first looks was approximated based on Raftery^53^. For this purpose, two generalized linear mixed-effects models were computed—one model of interest (model 1) and one null model (model 0). A BIC score was generated for each model (BIC_1_ and BIC_0_). To approximate the Bayes factor (*BF*_10_) the following formula was used: *BF*_10_ = exp [(BIC_0_ - BIC_1_)/2]. Note, that this computation differed from the computation of the Bayes factors for all other statistical tests.

The normality assumption of t-tests was tested using Shapiro–Wilk tests (function: *shapiro.test;* stats package version 3.5.2; R Core Team, 2018). Note that the assumption was violated for all t-tests, except for the dependent t-test. In cases in which the normality assumption was violated a robust version of the t-statistic was computed using the function *lmrob* of the *robustbase* package [version 0.93–6;^54^]. In terms of statistical significance, the results did not differ between the robust test and the preregistered standard t-test. Therefore, in the results section, only the results for the preregistered standard t-test are reported.

## Results

### Analyses of anticipatory looking measures

#### Between-participant analyses of first trials

**Figure 2:**
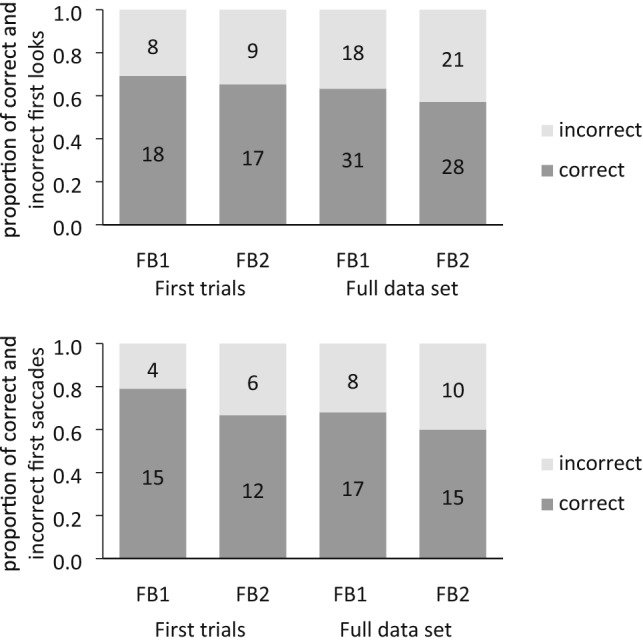
Proportion of correct first saccades (**a**) and correct first looks (**b**) in each condition (FB1, FB2) for the first trial and the full set analyses. Numbers written on the bars represent total numbers of observation.

**Figure 3:**
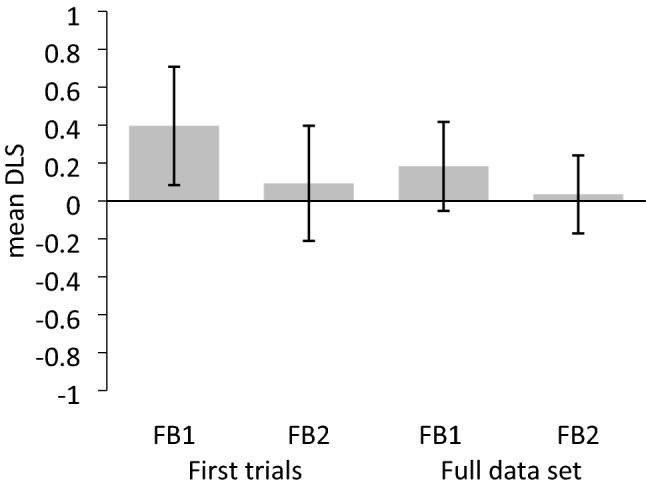
Mean differential looking score (DLS) in each condition (FB1, FB2) for the first trial and the full set analyses. Error bars depict 95% confidence intervals.

Figure 2 depicts the proportions of correct first saccades and first looks. Figure 3 depicts the mean DLS and 95% confidence intervals.

***First saccades.*** Of the 26 included participants that completed the FB1 condition as first trial, seven participants did not show a first saccade (i.e., movement of gaze), because they already looked at one AOI at sound cue onset. Similarly, of the 26 included participants that completed the FB2 condition as first trial, eight participants already looked at one AOI at sound cue onset. The number of correct first saccades was significantly above chance (0.5) in the FB1 condition (correct saccades *n* = 15, incorrect saccades *n* = 4, probability of success = 0.79, 95% CI_probability-of-success_ [0.54, 0.94], *p* = 0.019), but was not significantly different from chance (0.5) in the FB2 condition (correct saccades *n* = 12, incorrect saccades *n* = 6, probability of success = 0.67, 95% CI_probability-of-success_ [0.41, 0.87], *p* = 0.238, but note *BF*_10_ = 1.065). The number of correct first saccades did not significantly differ between the two conditions (*p* = 0.476, *OR* = 1.84, 95% CI_OR_ [0.34, 11.09], *BF*_10_ = 0.920).

***First looks.*** The number of correct first looks was neither significantly different from chance in the FB1 condition (correct looks *n* = 18, incorrect looks *n* = 8, probability of success = 0.69, 95% CI_probability-of-success_ [0.48, 0.86], *p* = 0.076, but note *BF*_10_ = 2.119), nor in the FB2 condition (correct looks *n* = 17, incorrect looks *n* = 9, probability of success = 0.65, 95% CI_probability-of-success_ [0.44, 0.83], *p* = 0.169, but note *BF*_10_ = 1.187). The number of correct first looks did not significantly differ between the two conditions (*p* = 1, *OR* = 1.19, 95% CI_OR_ [0.32, 4.47], *BF*_10_ = 0.630).

***DLS.*** Of the 26 included participants that completed the FB1 condition as first trial, one participant did not fixate on either AOI during the predetermined analysis interval. Therefore, DLS data for the first trial analysis of the FB1 condition was available for 25 participants. The mean DLS was significantly positive in the FB1 condition (*M* = 0.40, *SD* = 0.76, 95% CI [0.08, 0.71]), *t*(24) = 2.61, *p* = 0.015, *d* = 0.52, but it was not significantly different from zero in the FB2 condition (*M* = 0.09, *SD* = 0.75, 95% CI [− 0.21, 0.40]), *t*(25) = 0.63, *p* = 0.536, *d* = 0.12, *BF*_10_ = 0.248. The mean DLS did not significantly differ between the two conditions (FB1, FB2), *t*(49) = 1.43,* p* = 0.158, *d* = 0.40, 95% CI_difference-in-means_ [− 0.12, 0.73], *BF*_10_ = 0.649.

#### Full-set analyses including both trials

***First saccades. ***Analysing the full data set (i.e., the first and the second false-belief test trial for each included participant), the number of correct first saccades was significantly above chance (0.5) in the FB1 condition (correct saccades *n* = 24, incorrect saccades *n* = 9, probability of success = 0.73, 95% CI_probability-of-success_ [0.54, 0.87] , *p* = 0.014), but was not different from chance (0.5) in the FB2 condition (correct saccades *n* = 21, incorrect saccades *n* = 13, probability of success = 0.62, 95% CI_probability-of-success_ [0.44, 0.78], *p* = 0.230, *BF*_10_ = 0.867). Of all included participants, 25 participants showed a first saccade in both the FB1 and the FB2 condition and provided data for a within-participant comparison. The number of correct first saccades (correct FB1 *n* = 17, correct FB2 *n* = 15) did not significantly differ between the two conditions (FB1, FB2) within participants, χ^2^(1) = 1.57, *p* = 0.211, *BF*_10_ = 0.168.

***First looks.*** The number of correct first looks was significantly above chance (0.5) in the FB1 condition (correct looks *n* = 33, incorrect looks *n* = 18, probability of success = 0.65, 95% CI_probability-of-success_ [0.50, 0.78], *p* = 0.049), but was not different from chance (0.5) in the FB2 condition (correct looks *n* = 29, incorrect looks *n* = 21, probability of success = 0.58, 95% CI probability of success [0.43, 0.72], *p* = 0.322, *BF*_10_ = 0.584). A total of 49 participants showed a first look in both the FB1 and the FB2 condition and provided data for a within-participant comparison. The number of correct first looks (correct FB1 *n* = 31, correct FB2 *n* = 28) did not significantly differ between the two conditions (FB1, FB2) within participants, χ^2^(1) = 1.76, *p* = 0.185, *BF*_10_ = 0.122.

***DLS.*** Analysing the full data set, the mean DLS was neither significantly different from zero in the FB1 condition (*M* = 0.18, *SD* = 0.81, 95% CI [− 0.05, 0.42]), *t*(47) = 1.56, *p* = 0.126, *d* = 0.22, *BF*_10_ = 0.483, nor in the FB2 condition (*M* = 0.03, *SD* = 0.72, 95% CI [− 0.17, 0.24]), *t*(49) = 0.34, *p* = 0.737, *d* = 0.05, *BF*_10_ = 0.162. The DLS did not significantly differ between the two conditions (FB1, FB2) within participants (*M*_diff_ = 0.13, *SD*_diff_ = 1.04, 95% CI_mean-difference_ [− 0.18, 0.44]), *t*(45) = 0.87, *p* = 0.390, *d* = 0.13, *BF*_10_ = 0.228.

### Dropout Analyses

In this study, 52 participants were included in the data analysis, and 40 participants were excluded due to not passing the familiarization criterion defined by Senju et al. (^14,36^ i.e., longer looking to the correct AOI in the last familiarization trial). This dropout rate of 43%, was not significantly different from the dropout rate of 35% (20 participants included and 11 excluded due to not passing the familiarization criterion), in the study of Southgate, Senju^15^, *p* = 0.127, *BF*_10_ = 0.802. The dropout rate in this study was significantly higher than the dropout rate of 11% (17 participants included, two excluded due to the familiarization criterion) in the study of Senju, Southgate^36^. Compared to the two studies of Kulke, Wübker^35^ who tried to enhance the ecological validity of video stimuli, the dropout rate in this study was not different from the dropout rate of 37%, in study 1 (125 included, 72 excluded due to the familiarization criterion), *p* = 0.194, *BF*_10_ = 0.598. However, the dropout rate was significantly lower than the dropout rate of 56% in study 2 (125 included, 161 excluded due to the familiarization criterion), *p* = 0.015.

### Effects of guessing the aim

Of all 100 tested participants, three participants guessed the aim of the experiment, and only one participant reported that she was aware of the presence of a confederate. Considering those 52 participants that were included in the data analysis, two participants guessed the aim of the experiment, and no participant reported to be aware of the presence of a confederate. Of the two participants who guessed the aim, one participant started with the FB1 condition (DLS_FB1_ = 0.53, DLS_FB2_ = − 0.10, first look_FB1_ = incorrect, first look_FB2_ = incorrect) and one participant started with the FB2 condition (DLS_FB1_ = 1, DLS_FB2_ = 0.78, first look_FB1_ = correct, first look_FB2_ = incorrect). Both participants already fixated one AOI at sound cue onset in both false-belief trials, and therefore, no first saccades were coded.

### Exploratory comparison with previous studies

Descriptive statistics of first saccades and DLSs in previous studies, and the results of all statistical tests comparing the proportions of correct first saccades and the DLSs in this study with previous replication studies can be found in Table 1. In the following, only significant results are reported. The proportion of correct first saccades in the FB1 condition was significantly higher compared to study 3 of Kulke, Johannsen^33^, both when only the first trial was considered, *p* < 0.001, and when the full set of trials was considered, *p* < 0.001. Also, the proportion of correct first saccades in the FB2 condition was significantly higher compared to study 2 of Kulke, Wübker^35^, both when only the first trial was considered, *p* = 0.026, and when the full set of trials was considered, *p* = 0.007.

**Table 1:** Descriptive Statistics of first saccades and DLSs in previous studies and results of binomial tests (first saccades) and one-sample t-tests (DLSs) comparing the proportion of correct first saccades and the DLSs in this study to previous replication studies. The analysis either compares previous studies with the first trial of the current study (First) or with both trials (Full).

		First Saccades						DLS											
		FB1			FB2			FB1						FB2					
Comparison with	Analysis	% corr	*P*	*BF*_10_	% corr	*p*	*BF*_10_	*M (SD)*	95% CI	*p*	*t*	*df*	*BF*_10_	*M (SD)*	95% CI	*p*	*t*	*df*	*BF*_10_
Kulke, Johannsen, and Rakoczy^32^ (study 1)	First	.58	.101	1.065	.71	.795	0.555	0.09 (0.68)	[− 0.19, 0.37]	.058	1.99	24	1.143	0.38 (0.64)	[0.11, 0.64]	.066	− 1.92	25	1.014
	Full		.112	1.475		.257	0.692			.455	0.75	47	0.205			.002*	− 3.33	49	−
Kulke, Johannsen, and Rakoczy^32^ (study 3)	First	.27	< .001*	−	.67	1	0.522	− 0.13 (0.68)	[− 0.41, 0.15]	.002*	3.50	24	−	0.18 (0.75)	[− 0.13, 0.49]	.568	− 0.58	25	0.241
	Full		< .001*	−		.585	0.474			.010*	2.70	47	−			.168	− 1.40	49	0.384
Kulke, Reiß, Krist, and Rakoczy^28^ (study 2b^ab^)	First	.73	.797	0.693	.64	1	0.693	0.44 (0.59)	–	.796	− 0.26	24	0.218	0.23 (0.66)	–	.357	− 0.94	25	0.309
	Full		1	0.434		.858	0.416		–	.035*	− 2.17	47	−		–	.061	− 1.92	49	0.832
Kulke, von Duhn, Schneider, and Rakoczy^31^	First	.71	.615	0.693	.47	.104	1.408	0.53 (0.56)	[0.31, 0.76]	.382	− 0.89	24	0.302	− 0.26 (0.48)	[− 0.46, − 0.06]	.025*	2.39	25	−
	Full		1	0.445		.089	1.343			.005*	− 2.98	47	−			.006*	2.88	49	−
Kulke, Wübker, and Rakoczy^34^ (study 1)	First	.70	.464	0.715	.50	.238	1.065	0.67 (0.49)	[0.47, 0.88]	.079	− 1.84	24	0.899	− 0.10 (0.71)	[− 0.40, 0.19]	.197	1.33	25	0.454
	Full		.850	0.452		.230	0.867			< .001*	− 4.20	47	−			.185	1.34	49	0.358
Kulke, Wübker, and Rakoczy^34^ (study 2)	First	.70	.464	0.715	.39	.026*	−	0.28 (0.68)	[− 0.001, 0.56]	.457	0.76	24	0.273	0.02 (0.62)	[− 0.24, 0.28]	.627	0.49	25	0.232
	Full		.850	0.452		.007*	−			.401	− 0.85	47	0.220			.887	0.14	49	0.155

FB1 = false-belief condition 1; FB2 = false-belief condition 2*.* The proportion of correct first saccades in the FB1 condition of the current study was .79 (first trials) and .73 (full set of trials). The proportion of correct first saccades in the FB2 condition was .67 (first trials) and .62 (full set of trials). The mean DLS in the FB1 condition was 0.40, *SD* = 0.76, 95% CI [0.08, 0.71] (first trials) and 0.18, *SD* = 0.81, 95% CI [− 0.05, 0.42] (full set of trials). The mean DLS in the FB2 condition was 0.09, *SD* = 0.75, 95% CI [− 0.21, 0.40] (first trials) and 0.03, *SD* = 0.72, 95% CI [− 0.17, 0.24] (full set of trials).

^a^The data were taken from the adult sample in this study.

^b^For this study, no confidence intervals were reported.

*p < .05, two-tailed.

The mean DLS in the FB1 condition was significantly higher compared to study 3 of Kulke, Johannsen^33^, both when only the first trial was considered, *t*(24) = 3.50, *p* = 0.002, and when the full set of trials was considered, *t*(47) = 2.70, *p* = 0.010. Also, the mean DLS in the FB2 condition was significantly higher compared to the study of Kulke, von Duhn^32^, both when only the first trial was considered, *t*(25) = 2.39, *p* = 0.025, and when the full set of trials was considered, *t*(49) = 2.88, *p* = 0.006. However, when the full set of trials was considered, the mean DLS in the FB1 condition was significantly smaller compared to the mean DLSs in the study of Kulke, von Duhn^32^, *t*(47) = − 2.98, *p* = 0.005, study 2b of Kulke, Reiß^29^, *t*(47) =  − 2.17, *p* = 0.035, and study 1 of Kulke, Wübker^35^, *t*(47) =  − 4.20, *p* < 0.001. Considering the full set of trials the mean DLS in the FB2 condition was significantly smaller compared to study 1 of Kulke, Johannsen^33^, *t*(49) =  − 3.33, *p* = 0.002.

## Discussion

The current study aimed to investigate anticipatory looking in line with implicit ToM during realistic social interactions using mobile eye-tracking. Anticipatory looking behavior in line with ToM was found in the FB1 condition that is prone to alternative explanations but looking was at chance in the FB2 condition. The dropout rate due to participants not showing correct anticipatory looking during familiarization was high, comparable to previous studies. Correct anticipatory looking behavior was slightly less pronounced than in some previous studies [^29,32,33^study 1,^35^study 1] but more pronounced than in other previous studies^32,33^.

The current study found clear anticipatory looking when the belief-congruent location was identical to the last object location (FB1), but not when both locations differed, as in the FB2 condition. In the FB1 condition, ball tracking cannot be excluded as an alternative explanation to belief tracking. However, looking in FB2 did not significantly differ from FB1 and was not below chance, suggesting that ball tracking is not the only factor influencing the observed gaze pattern. Baillargeon, Buttelmann^2^ argue that chance performance in FB2 is sufficient to point towards the possibility of belief tracking, as gaze is similarly affected by ball tracking and belief tracking, leading to similar looking times to the belief-congruent and the last ball location in this condition. In line with their argumentation, the current study would provide evidence for some form of implicit ToM. This would suggest that in realistic social situations, implicit ToM can be measured more reliably than in previous replications using video stimuli in which gaze in the FB2 condition was mainly directed at the belief-incongruent location [e.g.,^26,29,32^]. However, in a more conservative view, the FB1 condition remains susceptible to alternative explanations^1^; in that sense, the study provides no clear evidence for implicit ToM and adds to the number of unclear findings and non-replications of anticipatory looking ToM tasks [e.g.^28,29,35^].

Exploratory comparisons of anticipatory looking measures with other studies suggest that the current study shows a clearer indication of implicit ToM than Study 3 by Kulke, Johannsen^33^ that removed confounds providing alternative explanations other than belief tracking, for example cueing by the turning direction of the actress and conditioning due to the ball always ending up in the same location. The current study also excluded these confounds. This suggests that if no alternative explanations exist, belief-tracking can somewhat be more clearly observed in the current real-life interaction study than in other studies using video stimuli. However, in paradigms that used stimuli containing the confounds of original studies, like the studies by Kulke, Reiß^29^, Kulke, von Duhn^32^, Kulke, Johannsen [^33^, study 1], Kulke, Wübker [^35^, study 1], effects were larger than in the current study. This, firstly, suggests that part of the effects in previous studies can be explained by confounds rather than by belief tracking. Secondly, the current findings are in between those of previous studies, suggesting that using a realistic interactive paradigm does not consistently lead to larger belief-tracking effects nor to smaller effects compared to the use of video stimuli.

The dropout rate in the current study was still high, as in previous studies^15,26,29,32,33,35,55^. This suggests that participants were not sufficiently more engaged by the real-life interaction to show reliable anticipatory looking patterns even during familiarization. This suggests that our initial assumption that engagement would improve due to real-life rather than video stimulation was not confirmed. Similar to previous studies, 43% of participants were excluded because they did not anticipatorily look to the correct side during familiarization. As this is almost half of the sample, gaze during familiarization may indeed be mainly related to chance, rather than reflecting true following of the scene^2^. Given that action anticipation is the cornerstone of anticipatory looking implicit ToM paradigms^1,2^, the absence of reliable action anticipation in familiarization trials calls into question whether the anticipatory looking measure is at all sufficiently reliable and valid to measure implicit ToM. If participants do not reliably show action anticipation in familiarization trials—in which no belief-tracking is required—there is no rational basis on which to interpret looking behavior in false-belief test trials as belief-based action anticipation [see e.g.,^1,2^]. The outcome measure used in this task, anticipatory looking behavior, may be affected by too many other factors making it unreliable for measuring belief tracking. The validity of anticipatory looking tasks is further questioned by a recent meta-analysis suggesting the existence of a publication bias in implicit ToM research, calling into question, whether initial findings indeed reflect true positives^3^.

The current study used mobile eye-tracking to investigate belief tracking in real social interaction situations. Previous studies suggest that participants gaze less towards faces when interacting live in the real world, compared to when watching video stimuli^38,39^. However, gaze patterns towards boxes were similar in the current study measuring live behavior as in studies using videos. This suggests that gaze towards locations that reflect other people’s beliefs is not affected by the live context as strongly as gaze towards other people’s faces. This fits well with the dual function of gaze hypothesis, suggesting that gaze is both used to *gather information* about the environment and to *signal to others* in social situations^42,43,56,57^. When one looks at faces of other people, this may be interpreted as a social signal, which is why participants may inhibit their gaze towards others’ faces in social situations when trying to avoid initiating a conversation. In contrast, gaze at objects (like the boxes in the current study) does not directly provoke social interaction. Therefore, real-life interactions might have larger effects on gaze towards other people than towards objects relevant to these people. Furthermore, gaze can not only be implicit but can also be consciously controlled. The anticipatory looking measures aim to measure implicit, incidental gaze. However, in the current situation, participants may have had various reasons to inhibit implicit gaze, for example they may have avoided gaze at the boxes to avoid providing cues for the confederate, or they may have looked at the boxes on purpose, to help the confederate with her task. These conscious gaze effects may have overshadowed potential implicit effects in the current task, as well as in the original video-based paradigm, leading to unreliable results.

It should be noted that the current study aimed to only alter the original anticipatory looking paradigms in one aspect: by live enacting it instead of presenting a video. This change did not improve the reliability of the task. However, it is possible that further adjustments may improve the paradigm. In particular, participants in the current task only observed the live interaction but were not involved in it themselves. Recent work suggests that the engagement in an action may significantly affect participants’ behavior^58^, and that simple observation of social situations significantly differs from social interaction^59^. Future research could therefore develop a comparable paradigm in which participants take part in the action themselves rather than only watching it. Furthermore, merely observing the interaction, participants had no direct benefit from inferring the protagonist’s mental states. If the protagonist’s actions have an immediate relevance to the participants’ goals, this could increase their motivation, leading to clearer anticipatory behavior^60^.

The current study is subject to some limitations. Firstly, although the confederate and the experimenter strictly adhered to the behavioral protocol, live-enacting the change-of-location task led to unsystematic natural variations in their behavior. This might have led to increased error variance compared to video-based studies, which might explain why the performance was not consistently increased compared to previous studies. Those effects of the live context that enhance anticipatory looking behavior may be canceled out due to decreases in effects due to natural variations. Secondly, this study implemented some changes to the set-up in the original video stimuli. For instance, this study did not use a panel with windows and therefore no windows were illuminated. This might have reduced action anticipation, as participants’ looks were not automatically cued to either AOI. Note however, that the changes are in line with other conceptual replication studies^33^. Thirdly, 8 of the 100 participants, 5 of whom were included, reported during the debriefing procedure that they noticed that the confederate waited for some time before reaching for the object. Because of this relatively small number we are confident that this did not fundamentally influence the results. However, since the delay in reaching was modelled after the original video stimuli, this might also be an issue with previous replication attempts using video stimuli. Future studies should investigate whether anticipatory looking behavior becomes evident more reliably with a shorter and more realistic delay in reaching. Lastly, to increase statistical power, each participant completed both false-belief trials in a within-participant design. Within-participant designs have been used in previous video-based studies, with no significant changes in gaze behavior occurring with trial number^32^. However, in the realistic scenario in the current study, after the first false-belief trial, there might have been uncertainty whether the confederate expected to be deceived in the second false-belief trial and therefore might intentionally look for the object under the wrong box. Based on this, the first trial analysis may have been more reliable, compared to the full set analysis. As effects in the current study were overall reduced for the analysis including both trials rather than just the first one, future research should avoid repetition of trials in live situations. It should be noted that in the current study we focused on implicit ToM. We decided not to include any explicit measures because we wanted participants to remain blind to the aim of the study. However, a previous set of implicit ToM studies tested adult participants with an implicit paradigm and subsequently asked them explicitly about the belief of the protagonist in the video presented to them. Although the implicit measure did not provide evidence for correct anticipatory looking, 100% (25 of 25) of participants correctly answered the explicit question both in the FB1 and the FB2 condition^35^. This suggests that implicit and explicit measures in simple video-based paradigms may be unrelated. Other studies have shown negligible relations between implicit and explicit measures^25,61^. However, future research could explore whether relations between implicit anticipatory looking measures and more fine-grained explicit measures (e.g. measured using the “reading mind in the eyes” test^62^) exist.

In summary, the current study did not find clear evidence of implicit ToM using an anticipatory looking paradigm even under very realistic circumstances using real-life mobile eye-tracking. This suggests that even if implicit ToM exists, it is very difficult to detect and anticipatory looking may not be a suitable measure to demonstrate it.

## Supplementary information

Supplementary Information A.

Supplementary Information B.

Supplementary Information C.

Supplementary Information D.

## Data Availability

The study has been preregistered with the Open Science Framework and the full data and analysis scripts are openly shared alongside the manuscript.
