# Supplementary material for: Implicit Theory of Mind under realistic social circumstances measured with mobile eye-tracking
Source: Sci Rep. 2021 Jan 13;11:1215. doi: 10.1038/s41598-020-80614-5 (PMC7806733; doi:10.1038/s41598-020-80614-5)
Supplement: Supplementary file 1 — Supplementary Information A. [file 41598_2020_80614_MOESM1_ESM.docx]

Supplement A. Set-Up

**Implicit Theory of Mind under realistic social circumstances measured with mobile eye-tracking**

**Louisa Kulke & Max Andreas Bosse Hinrichs**

The set-up of this study is depicted in Figure 1. Testing took place in a room with white walls and one window. Curtains were closed, and the light was turned on to allow for approximately constant lighting conditions across participants. Wearing the mobile eye-tracker, participants were seated on one side of a white testing table (H: 72 cm, L: 60 cm, W: 80), facing the confederate sitting behind a wooden curtain frame (H: 161 cm, W: 130 cm) on the other side of the table (Figure 1a). The experimenter was sitting on a small stool (H: 44.5 cm, L: 31.5 cm, W: 31.5 cm) at the participant’s left side. A black blind (H: 195 cm, W: 120 cm) was attached to the wooden curtain frame. The blind was equipped with a white adjusting rod (L: 75 cm when the blind was open, L: 103 cm when the blind was closed) which was placed on the experimenter’s side and allowed the experimenter to open and close the blind while staying seated (Figure 1b). A yellow spikey massage ball (diameter of 7 cm) served as the object for our change-of-location task (Figure 1). Two plain green boxes (H: 13.8 cm, L: 13.5 cm, W: 13.5 cm) were placed 26 cm apart from each other (see Figure 1b) and served as containments.
 In addition to the blind, three controls were implemented to ensure that participants believed that the confederate did not witness the object-removal in false-belief test trials. Firstly, a black felt mat (80 cm x 54 cm) on the table served to attenuate sound so that the confederate could not hear the experimenter lifting the boxes while the blind was closed (Figure 1). Secondly, a black board (119 cm x 60 cm) was attached to the lower front side of the curtain frame to avoid the confederate from seeing shadows beneath the blind (Figure 1a). Lastly, an additional curtain was attached to the experimenter’s side of the curtain frame to ensure that the confederate could not see the experimenter past the side of the curtain frame. For each box, two white markers (i.e., adhesive tape stripes, 1.5 cm x 0.7 cm) marked the location of the boxes. A white cross (3.5 cm x 3.5 cm) central between the two boxes marked the ball location at the beginning of each trial. The cue sound was activated manually by the experimenter using an iPhone SE (Apple, iPhone model: A1723, volume set to maximum). The iPhone was lying on a box (H: 44.5 cm, L: 23cm, W: 11.5 cm) to the left of the experimenter’ stool (i.e., the side facing the blind) not visible for the participant. The backside of the participants’ chair was placed at 134 cm from the front side of the boxes (i.e., the side facing the participant, see Figure 1a) and at 241.5 cm from the backside of the confederate’s chair (see Figure 1a). For each test session, the confederate and the experimenter wore the same clothing. The experimenter wore black jeans and a plain white t-shirt. The confederate wore black jeans and a long-sleeved dark-grey shirt.

Two webcams (c920 Logitech HD Pro Webcams, full HD 1080p, equipped with a built-in stereo microphone) were placed behind the participant’s chair (i.e., out of sight for the participant) to record the behavior of the experimenter and the confederate (see Figure 1a). One webcam (Figure 1, Webcam 1) was mounted on a camera stand slightly to the right side of (but still behind) the participant’s chair and was connected to a Dell Latitude E6510 computer (operating system: Windows 8.1 Enterprise, processor type: Intel Core i5-580M 2.67 GHz, RAM: 3.00 GB, system type: 64-bit operating system). The other webcam (Figure 1, Webcam 2) was attached on a wall slightly to the left of the participant’s chair and was connected to a Dell Latitude E5530 computer (operating system: Windows 8.1. Enterprise, processor type: Intel Core i5-3210M 2.50 GHz, RAM: 4.00 GB, system type: 64-bit operating system).

a)

b)


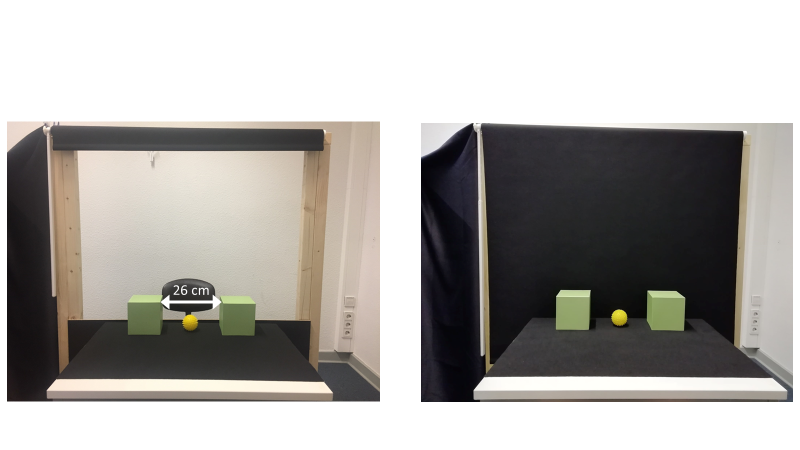

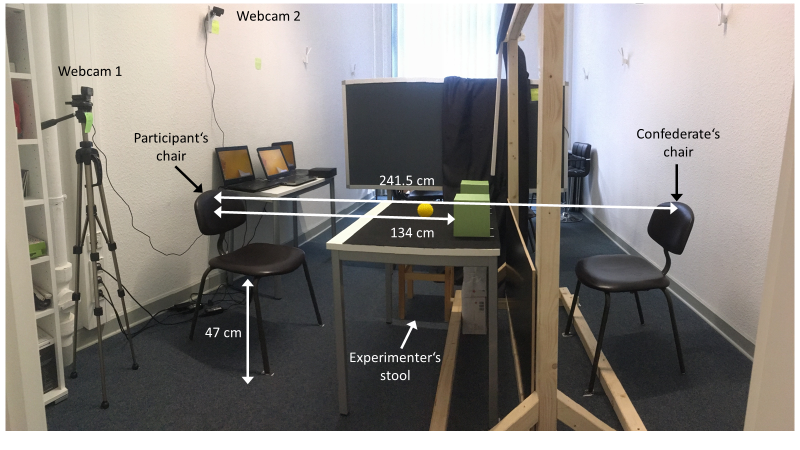


*Figure 1. a)* Panoramic view of the set-up with equipment labelled and distances reported in cm. b) view from the participant’s chair when the blind was open (left) and when the blind was closed (right).
