# Supplementary material for: Implicit Theory of Mind under realistic social circumstances measured with mobile eye-tracking
Source: Sci Rep. 2021 Jan 13;11:1215. doi: 10.1038/s41598-020-80614-5 (PMC7806733; doi:10.1038/s41598-020-80614-5)
Supplement: Supplementary file 2 — Supplementary Information B. [file 41598_2020_80614_MOESM2_ESM.docx]

**Supplement B. Areas of Interest Used for Eye Tracking Data Reduction**

**Implicit Theory of Mind under realistic social circumstances measured with mobile eye-tracking**

**Louisa Kulke & Max Andreas Bosse Hinrichs**

*
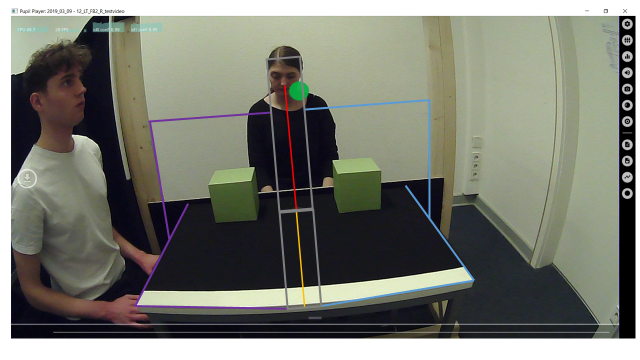
*
*Figure 1*. Graphical illustration of the two areas of interest (AOIs) used in this study. Purple lines depict the outer borders of the left AOI, and blue lines depict the outer borders of the right AOI. A central corridor separates both AOIs (grey). The position of the central corridor was defined separately for the area on the table and the area within the curtain frame. An imaginary line (red) drawn from the center of the confederate’s nose to the center of a white cross central between the two boxes defined the position of the central corridor within the curtain frame. An imaginary line (yellow) drawn from the white cross central between the two boxes to the center of the front edge of the table defined the position of the central corridor on the table. A box with the width of the confederate’s head (i.e., ear to ear distance) around the two lines marked the central corridor within the curtain frame and on the table, respectively. The green circle represents the “gaze circle”, which shows where a participant looked at in a respective frame.
